# Supplementary material for: Autoinducer-2 and bile salts induce c-di-GMP synthesis to repress the T3SS via a T3SS chaperone
Source: Nat Commun. 2022 Nov 5;13:6684. doi: 10.1038/s41467-022-34607-9 (PMC9637222; doi:10.1038/s41467-022-34607-9)
Supplement: Supplementary file 1 — Supplementary Information [file 41467_2022_34607_MOESM1_ESM.pdf]

Supplementary Information

Autoinducer-2 and bile salts induce c-di-GMP synthesis to repress the T3SS via a T3SS chaperone

This PDF file includes:

Supplementary Figures 1-26

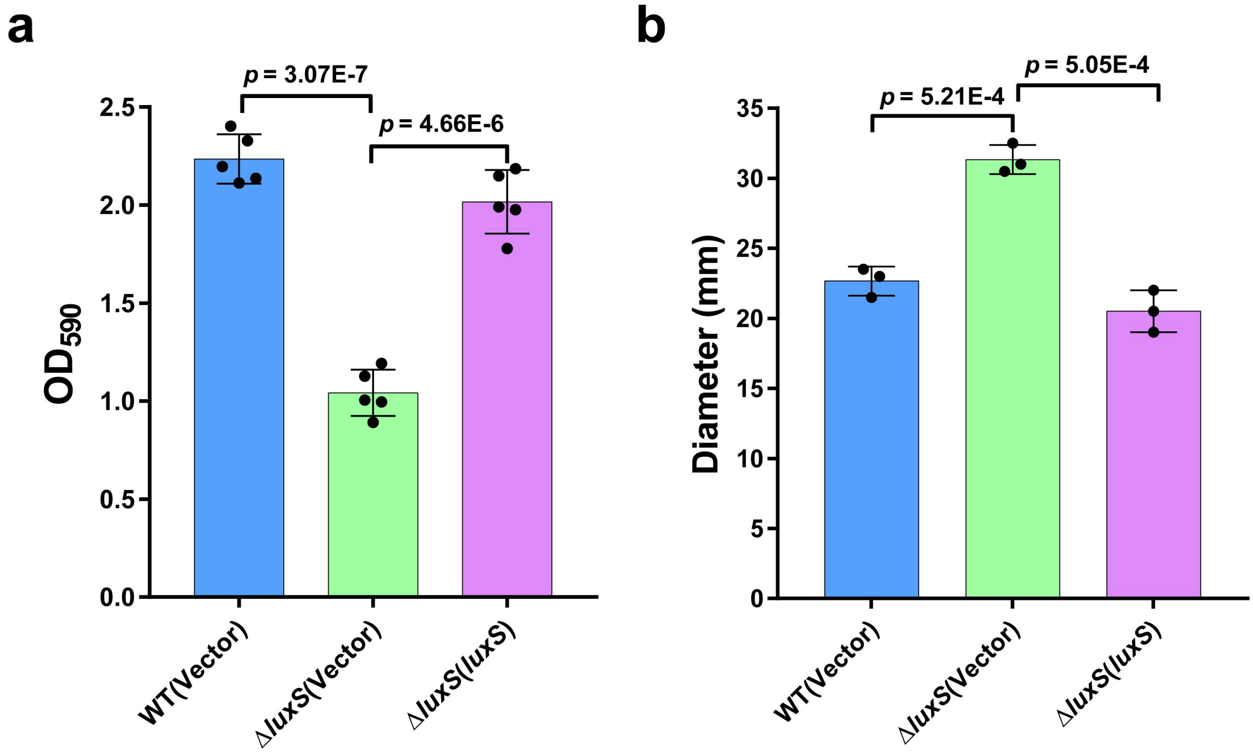

Supplementary Fig. 1 AI-2 regulates biofilm formation and swimming motility in *S.* Typhimurium
a 200 µl aliquots of *S.* Typhimurium strains with an OD_{600} of 0.05 in LB medium were placed into the wells of a 96-well plate. After incubation at 37 °C for 24 h, biofilms were stained with crystal violet and quantified using optical density measurement. Data are mean ± s.e.m. of five independent experiments. **b** 2 µl aliquots of *S.* Typhimurium strains with an OD_{600} of 0.5 in LB medium were spotted onto 0.3% tryptone agar. After incubation at 37 °C for 12 h, motility halos were measured. Data are mean ± s.e.m. of three independent experiments. **a, b** Statistical significance was determined using two-tailed unpaired Student's *t*-test and $p < 0.05$ was considered statistically significant. Source data are provided as a Source Data file.

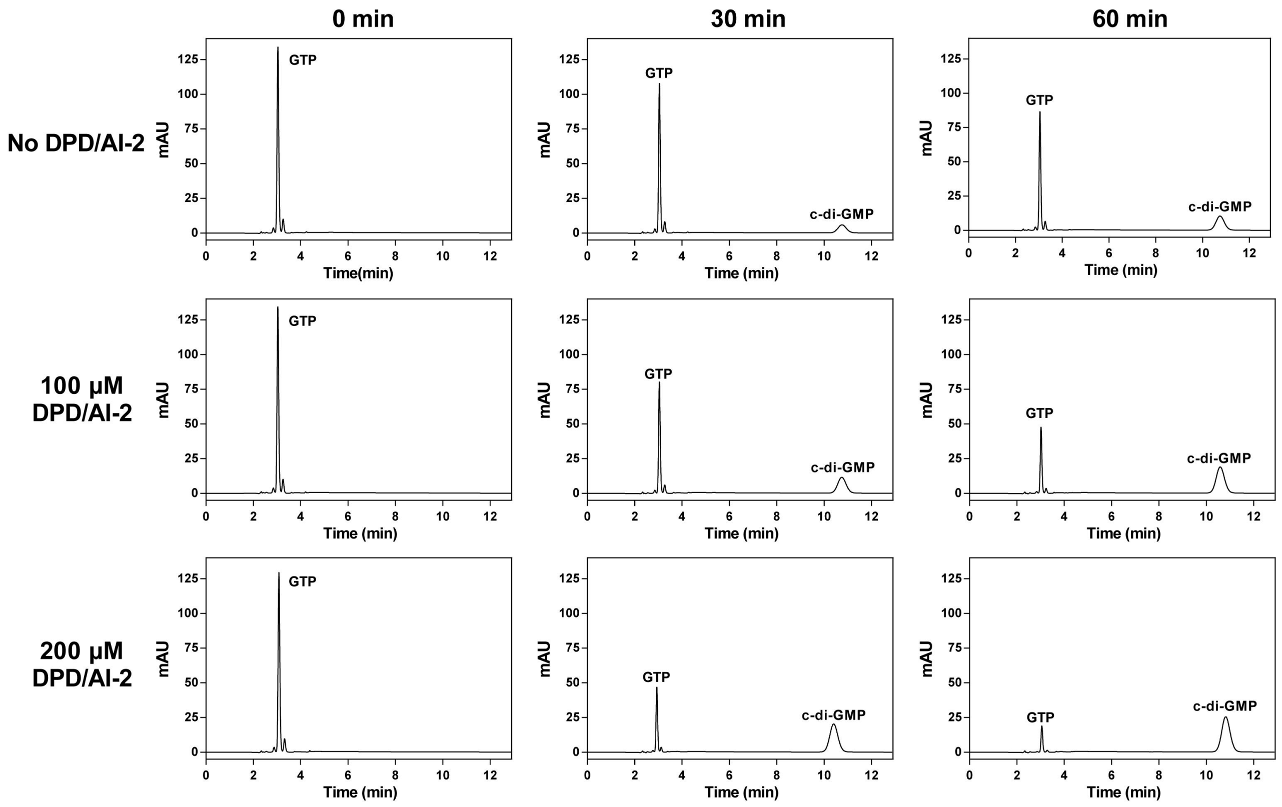

Supplementary Fig. 2 AI-2 induces the DGC activity of YeaJ in c-di-GMP synthesis

Membrane fractions containing full-length YeaJ were incubated with GTP in the presence or absence of DPD/AI-2 at 30°C for 0, 30 and 60 min and the products were analyzed by HPLC. HPLC spectra shown are representatives of three independent experiments with similar results.

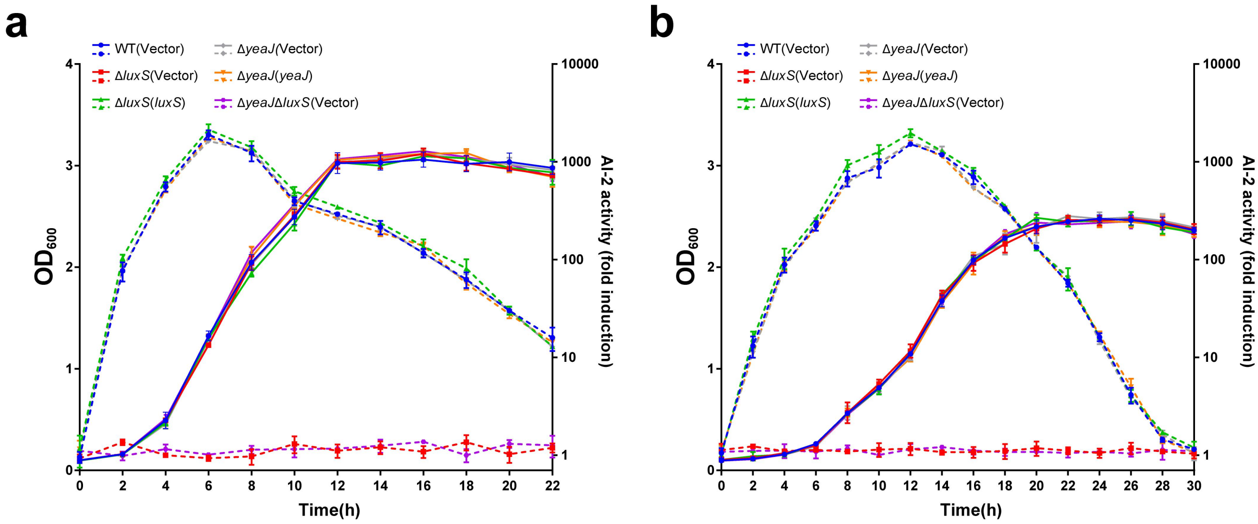

Supplementary Fig. 3 Growth curves and extracellular AI-2 activities of *S*. Typhimurium strains

S. Typhimurium strains were grown at 37 °C in LB medium with shaking (**a**) or in modified LB medium containing 0.3 M NaCl without agitation (**b**). At each time point during growth, cell density was recorded by measuring the optical density at 600 nm (solid line) and cell supernatants were collected, filtered, and measured for AI-2 activity by using the *V. harveyi* MM32 reporter assay (dashed line). Data are mean ± s.d. of three biological replicates. Source data are provided as a Source Data file.

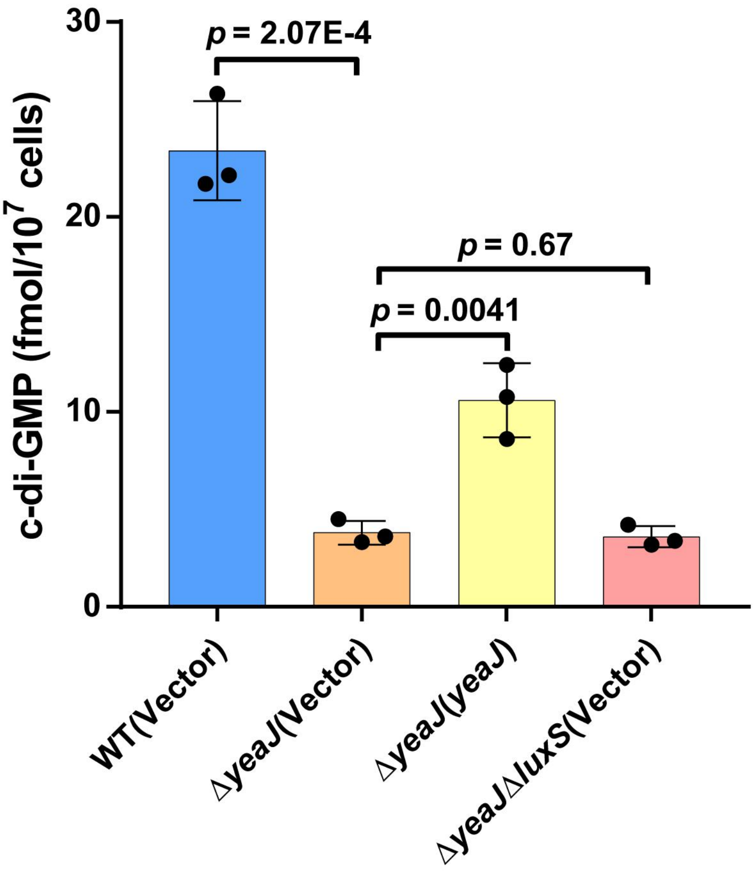

Supplementary Fig. 4 Deletion of *luxS* did not alter intracellular levels of c-di-GMP in a Δ*yeaJ* background

LC-MS/MS measurements of cellular levels of c-di-GMP in *S.* Typhimurium strains. All strains were grown in LB broth at 37 °C with shaking to an OD_{600} of 1.3 (Supplementary Fig. 3a), and then 15 ml aliquots of the bacterial cultures were subjected to nucleotide extractions. Cellular levels of c-di-GMP were quantified by LC-MS/MS analysis. Data are mean ± s.d. of three biological replicates. Statistical significance was evaluated using the two-tailed unpaired Student's *t*-test. *P* values < 0.05 indicate significant differences. Source data are provided as a Source Data file.

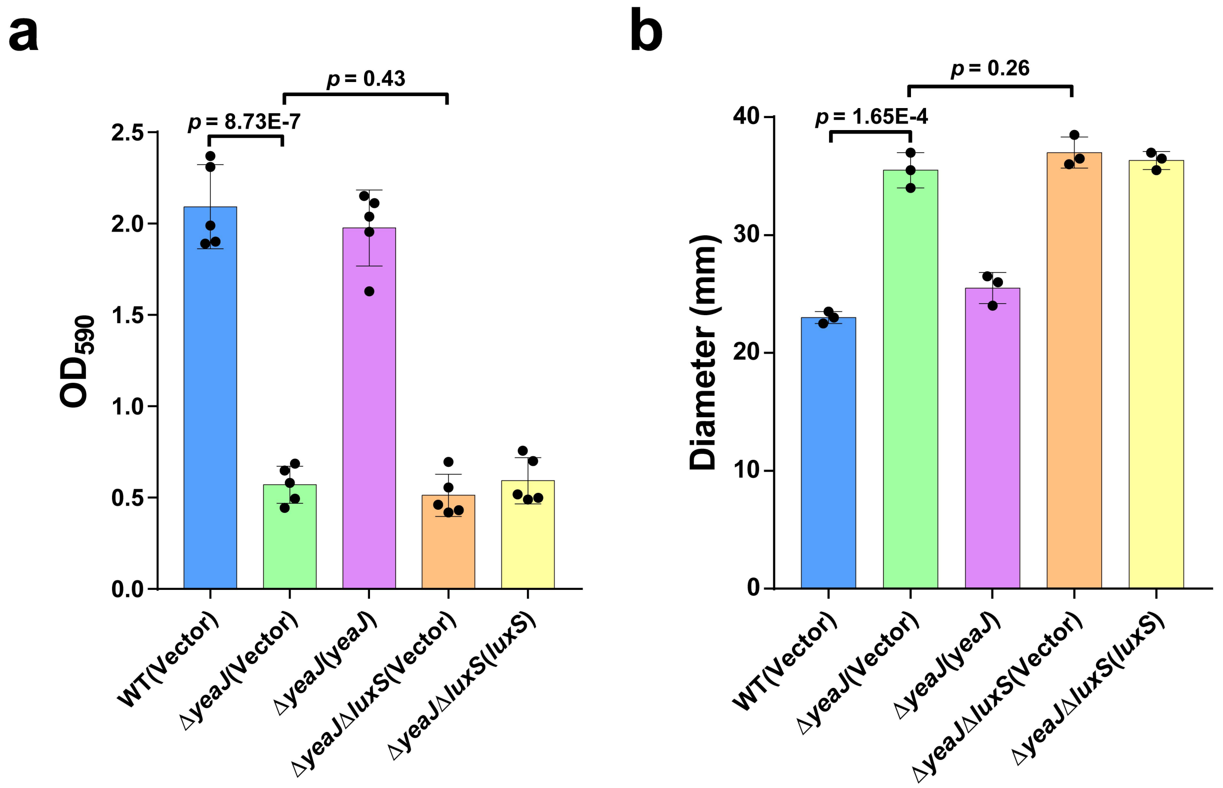

Supplementary Fig. 5 AI-2-mediated regulation of biofilm formation and motility in *S.* Typhimurium is dependent on the DGC YeaJ

a Biofilm formation by *S.* Typhimurium strains was measured using a crystal violet staining method as described in Supplementary Fig. 1a. Data are mean ± s.e.m. of five independent experiments. **b** *S.* Typhimurium strains were measured for swimming motility as described in Supplementary Fig. 1b. Data are mean ± s.e.m. of three independent experiments. **a, b** Statistical significance was determined by the two-tailed unpaired Student's *t*-test and $p < 0.05$ was considered to be statistically significant. Source data are provided as a Source Data file.

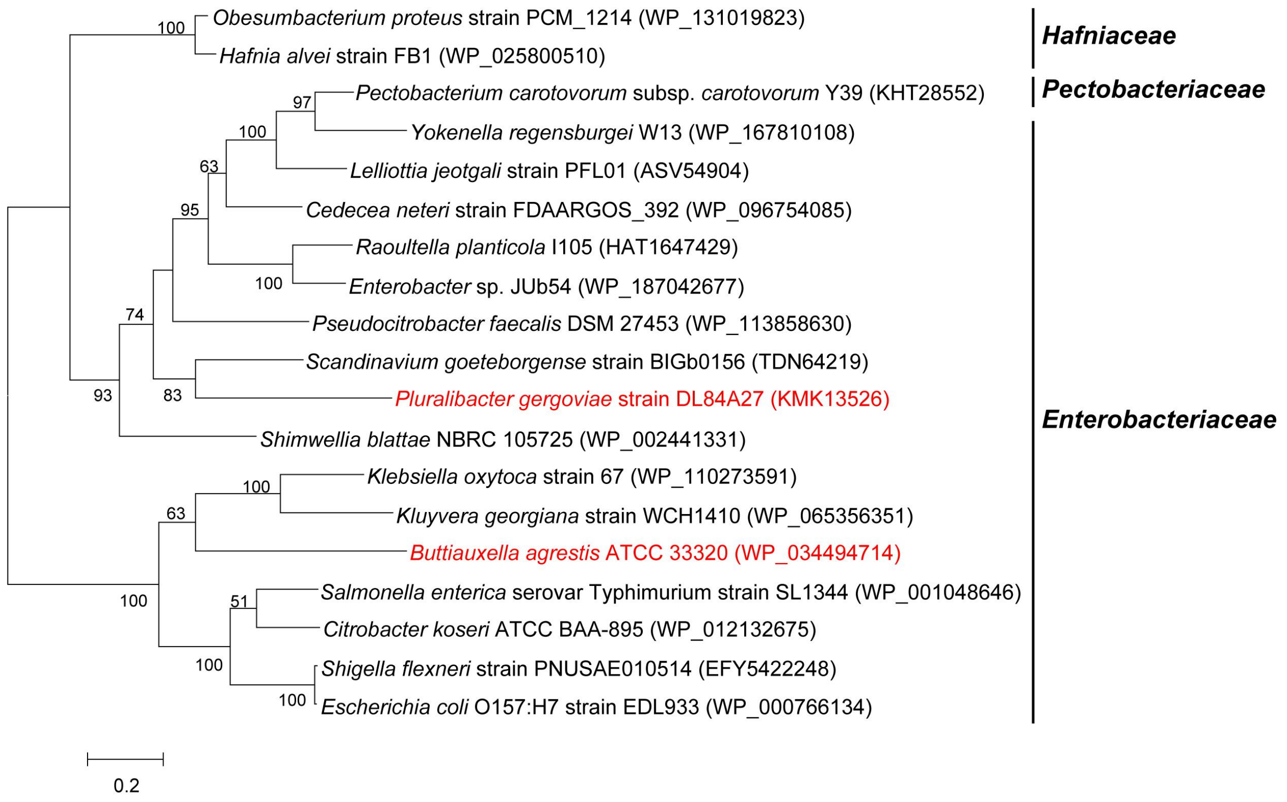

Supplementary Fig. 6 Maximum-likelihood phylogenetic tree showing the relationship of the GAPES1 domain-containing diguanylate cyclase YeaJ from *S.* Typhimurium and 18 high-confidence YeaJ orthologs from other genera

High-confidence YeaJ homologs were searched by BLASTP and further identified using InterProScan 5 against the Pfam 34.0 database (Supplementary Data 1). One representative sequence for each genus was randomly selected and multiple sequence alignments were performed with clustalX version 1.81. The phylogenetic tree was reconstructed by using the maximum-likelihood method based on the Jones-Taylor-Thornton (JTT) model embedded in the MEGA7 software. The bacterial species to which YeaJ homologs belong are provided and the NCBI accession number for each YeaJ homolog is given in parentheses. The YeaJ homologs whose GAPES1 domains showed no detectable AI-2 binding activity are indicated in red. Phyletic distribution of the 19 GAPES1-containing proteins at the family level is shown. Bootstrap values (expressed as percentages of 1000 replications) greater than 50% are shown on the branch. Scale bar indicates evolutionary distance of 0.2 amino acid substitutions per position.

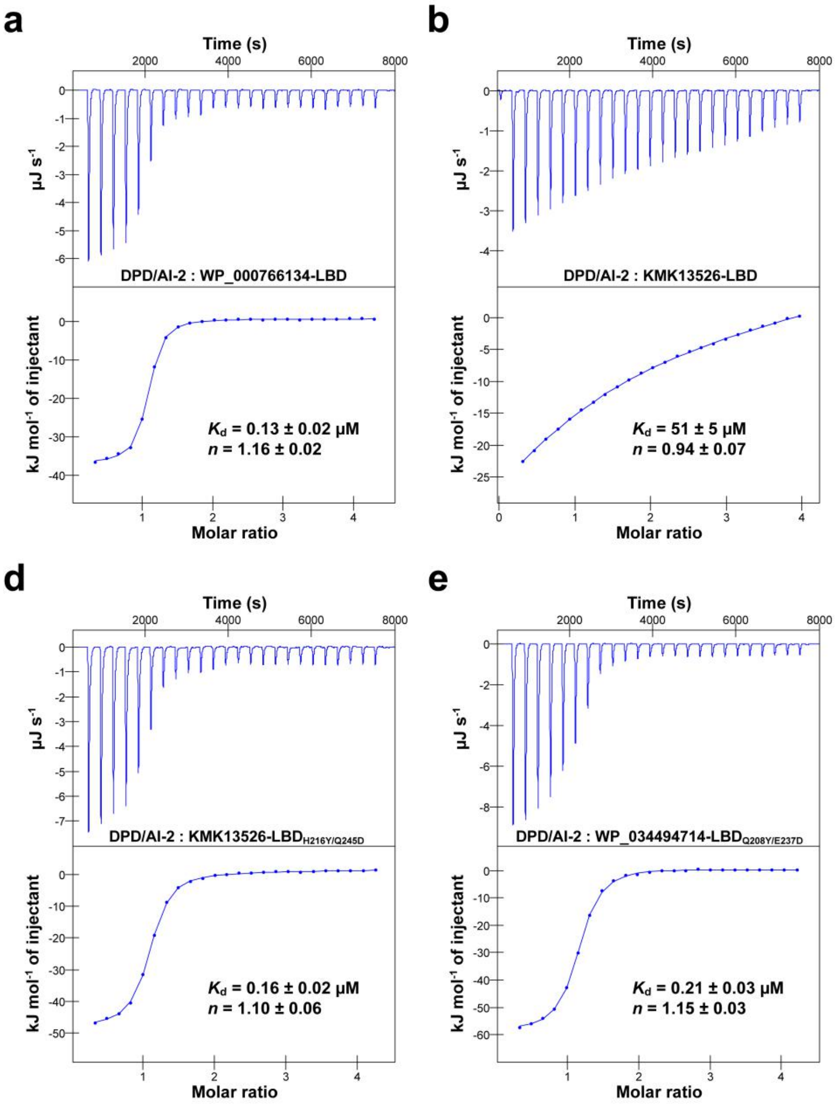

Supplementary Fig. 7 Isotherms representing binding of the wild type or mutant GAPES1 domains of YeaJ homologs with DPD/AI-2 as measured by ITC

ITC data and plots of injected heat for injections of 700 μM DPD/AI-2 into the sample cell containing 70 μM WP_000766134-LBD (**a**), KMK13526-LBD (**b**), WP_034494714 (**c**), KMK13526-LBD$_{H216Y/Q245D}$ (**d**) or WP_034494714$_{Q208Y/E237D}$ (**e**) are shown in the upper and lower plots, respectively. The heats of ligand dilution were subtracted from the heats of injection and the corrected data were fit to a one-site binding model to determine the K_d values and binding stoichiometry (n) by the NanoAnalyze software. Isotherms shown are one representative of three independent experiments with similar results, and the K_d and n values are presented as mean ± s.d. of the three independent experiments. Source data are provided as a Source Data file.

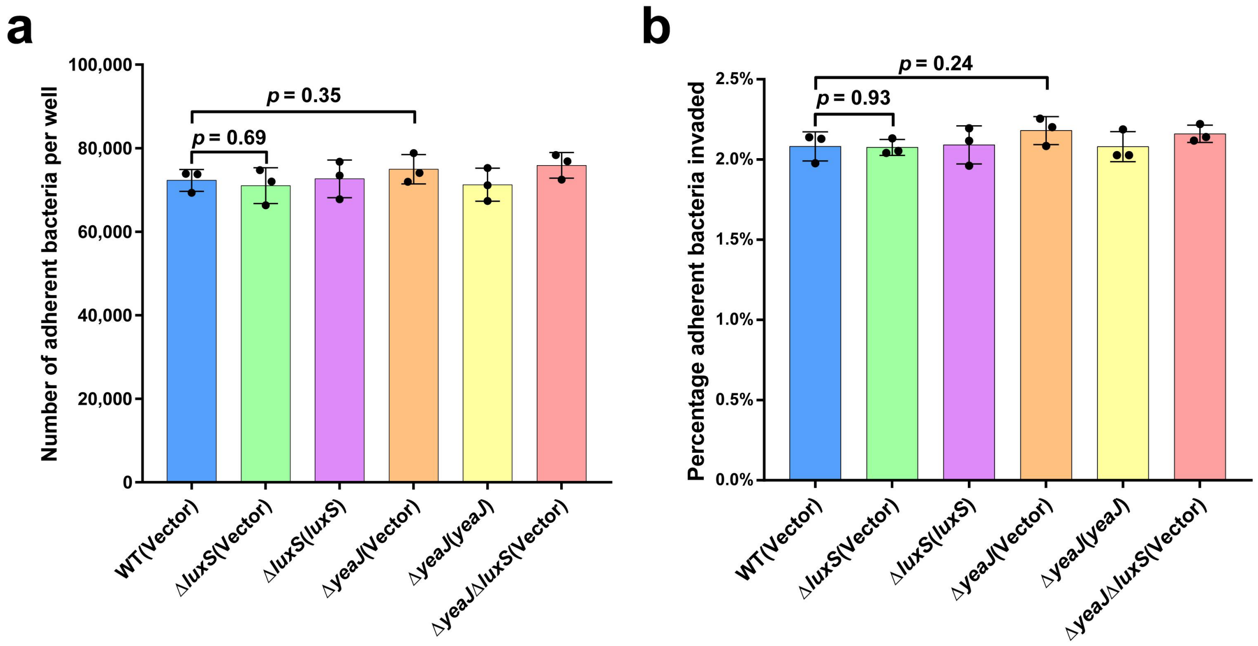

Supplementary Fig. 8 Adherence to and invasion of Caco-2 cells by S. Typhimurium cultures grown in LB broth with agitation until log phase when the AI-2 concentration in wild-type culture supernatant is maximal

S. Typhimurium strains were grown to an OD_{600} of 1.3 (Supplementary Fig. 3a), and then bacterial cells were collected, washed and resuspended in PBS. Monolayer cultures of Caco-2 cells at a density of 1 × 10^5 cells per well were infected with bacterial cultures at a multiplicity of infection (MOI) of 50. Bacterial adherence (**a**) was measured by CFU counting and presented as the total number of cell-associated bacteria per well, and bacterial invasion (**b**) was expressed as a percentage of intracellular bacterial counts relative to the total adherent bacteria. Data are mean ± s.e.m. of three independent experiments. Statistical significance was determined by two-tailed Student's *t*-test. $P < 0.05$ indicates a statistically significant difference. Source data are provided as a Source Data file.

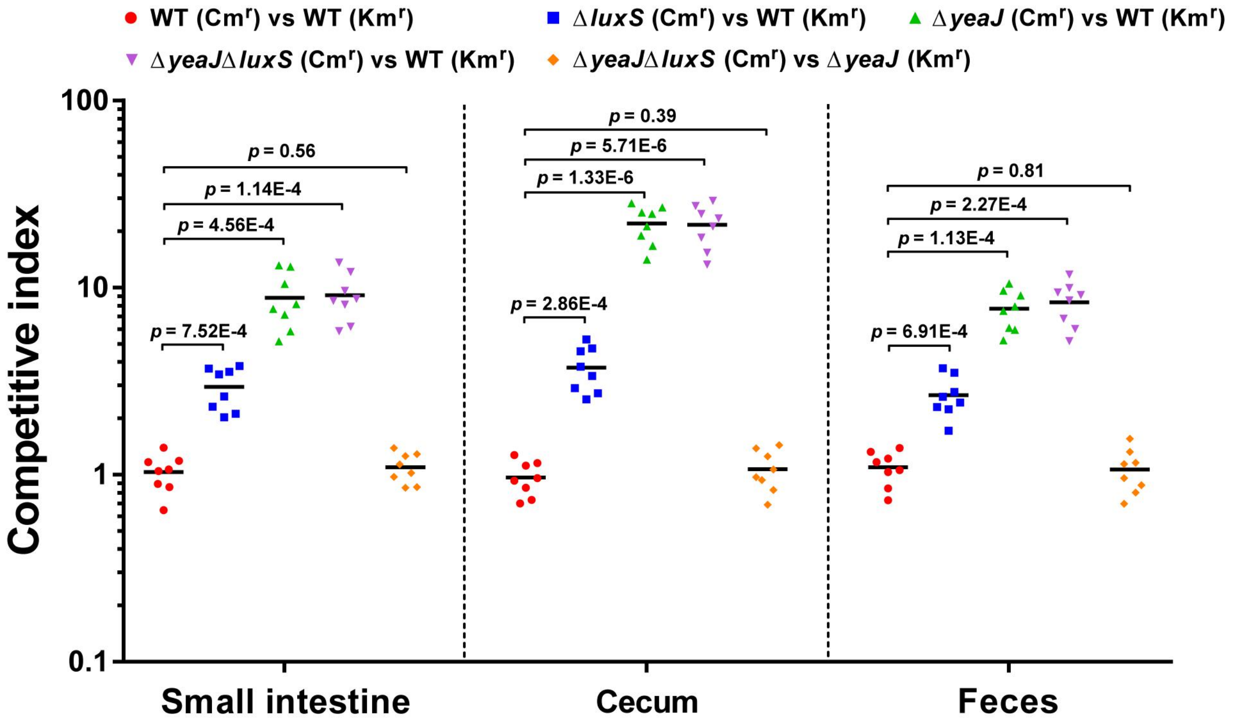

Supplementary Fig. 9 Competition assays in mice between *S*. Typhimurium strains

S. Typhimurium cultures grown in modified LB medium containing 0.3 M NaCl at 37 °C without agitation to an OD_{600} of 1.1 were collected, washed and resuspended in PBS. BALB/c mice pre-treated with streptomycin were orally gavaged with a 1:1 mixture of two *S*. Typhimurium strains (5 × 10^8 CFU for each) carrying chloramphenicol-resistant (Cm^r) pBBR1MCS1 and kanamycin-resistant (Km^r) pKT100, respectively. Feces, cecum and small intestine tissues harvested at day 2 post infection were homogenized, serially diluted and plated on antibiotic selective LB agar plates for CFU enumeration. CI values were calculated as the ratios of the test strains carrying pBBR1MCS1 versus (vs) the control strains carrying pKT100 recovered from mice. Horizontal lines represent the geometric mean CI value for each group (n = 8 mice per group). Statistical significance was determined with a two-tailed Mann–Whitney *U* test. A p value less than 0.05 was considered to be statistically significant. Source data are provided as a Source Data file.

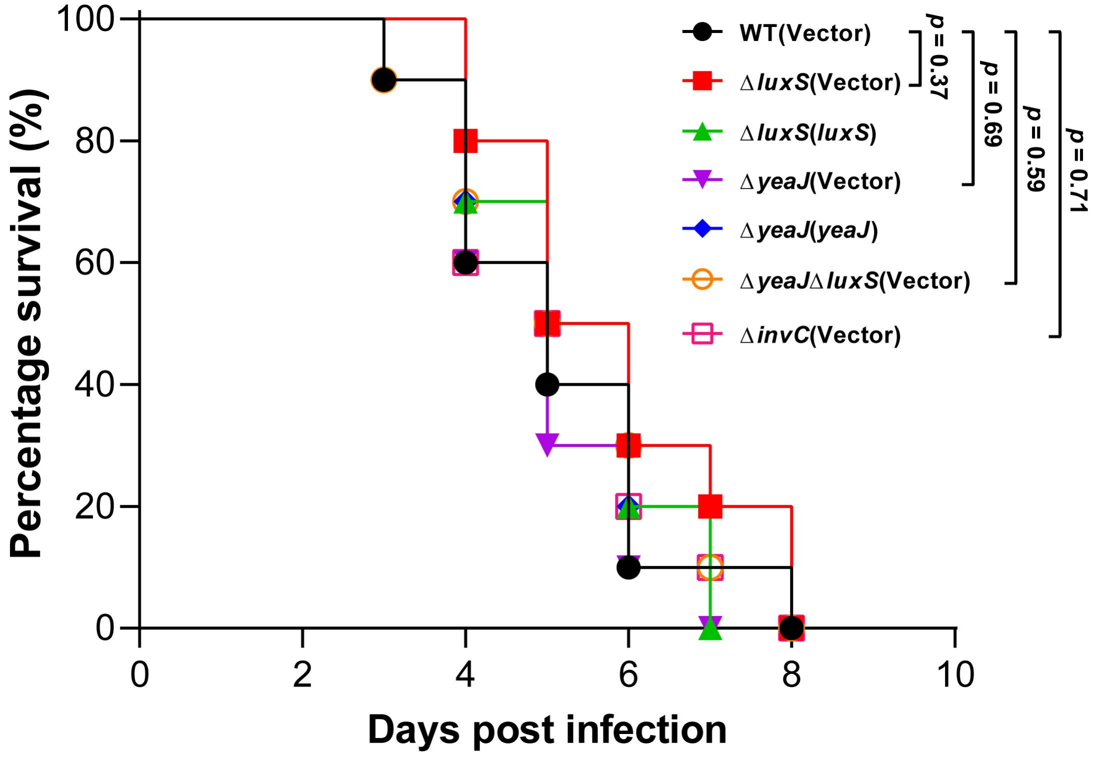

Supplementary Fig. 10 Survival curves of mice infected intraperitoneally with *S*. Typhimurium strains

S. Typhimurium strains were grown in modified LB medium containing 0.3 M NaCl at 37 °C under static conditions to an OD_{600} of 1.1, and then bacterial cells were collected, washed and resuspended in PBS. BALB/c mice were injected intraperitoneally with 10^4 CFU of each *S*. Typhimurium strain in 100 μl of PBS and the mortality of infected mice was recorded daily. Data are illustrated as percentage of mice survival (n = 10 mice per group). Statistical significance was determined using Log-rank (Mantel–Cox) test. Differences were considered statistically significant at $p < 0.05$. Source data are provided as a Source Data file.

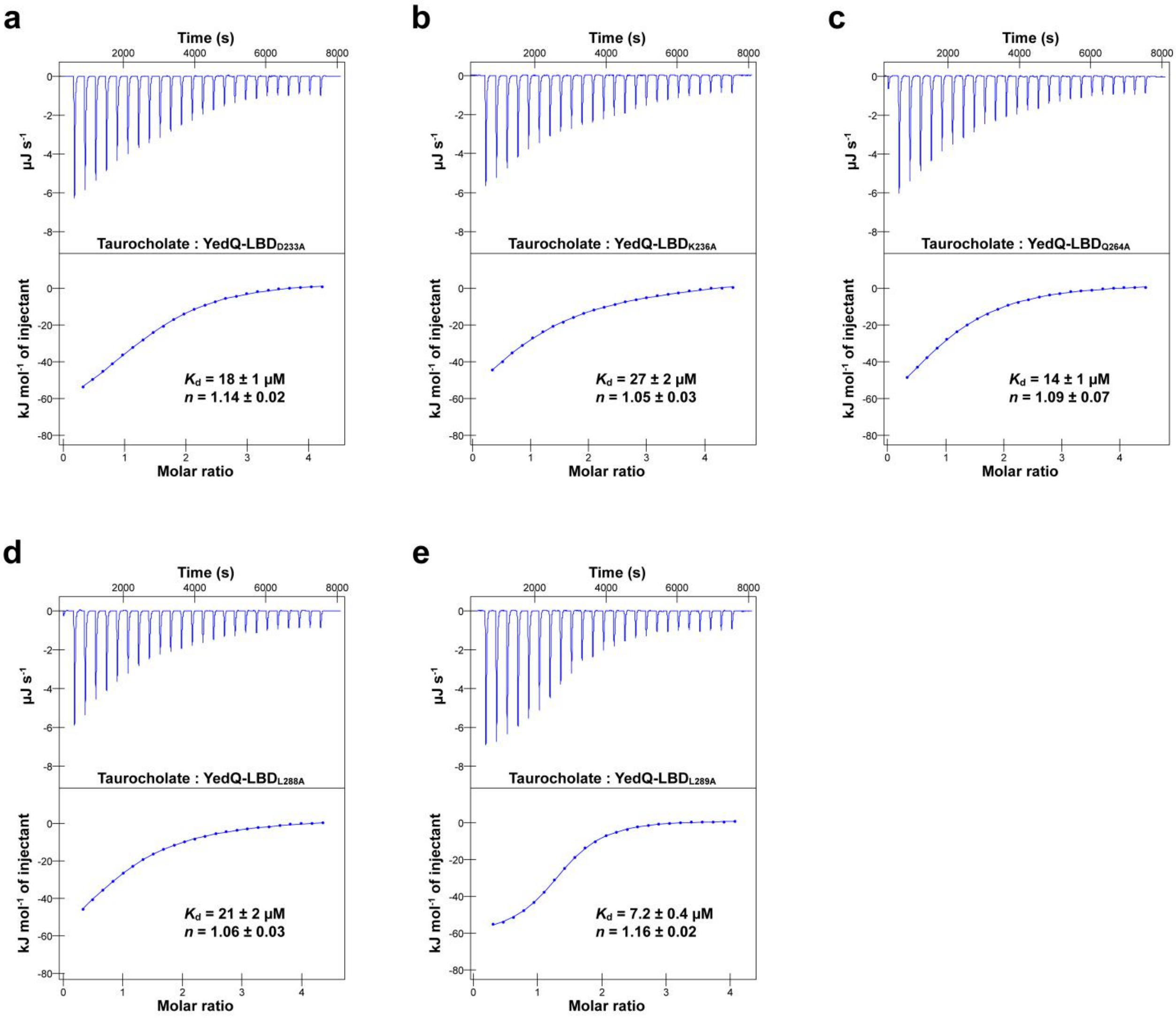

Supplementary Fig. 11 Five mutants of YedQ-LBD show lower binding affinity to taurocholate compared with the wild type

The binding affinity was evaluated using ITC analysis. ITC data and plots of injected heat for injections of 100 μM taurocholate into the sample cell containing 10 μM D233A (**a**), K236A (**b**), Q264A (**c**), L288A (**d**) or L289A (**e**) mutants of YedQ-LBD are shown in the upper and lower plots, respectively. The binding curves were corrected for the dilution effects in the final analysis. Data shown are one representative of three independent experiments with similar results. The K_d and binding stoichiometry (n) values were presented as mean ± s.d. of three independent experiments. Source data are provided as a Source Data file.

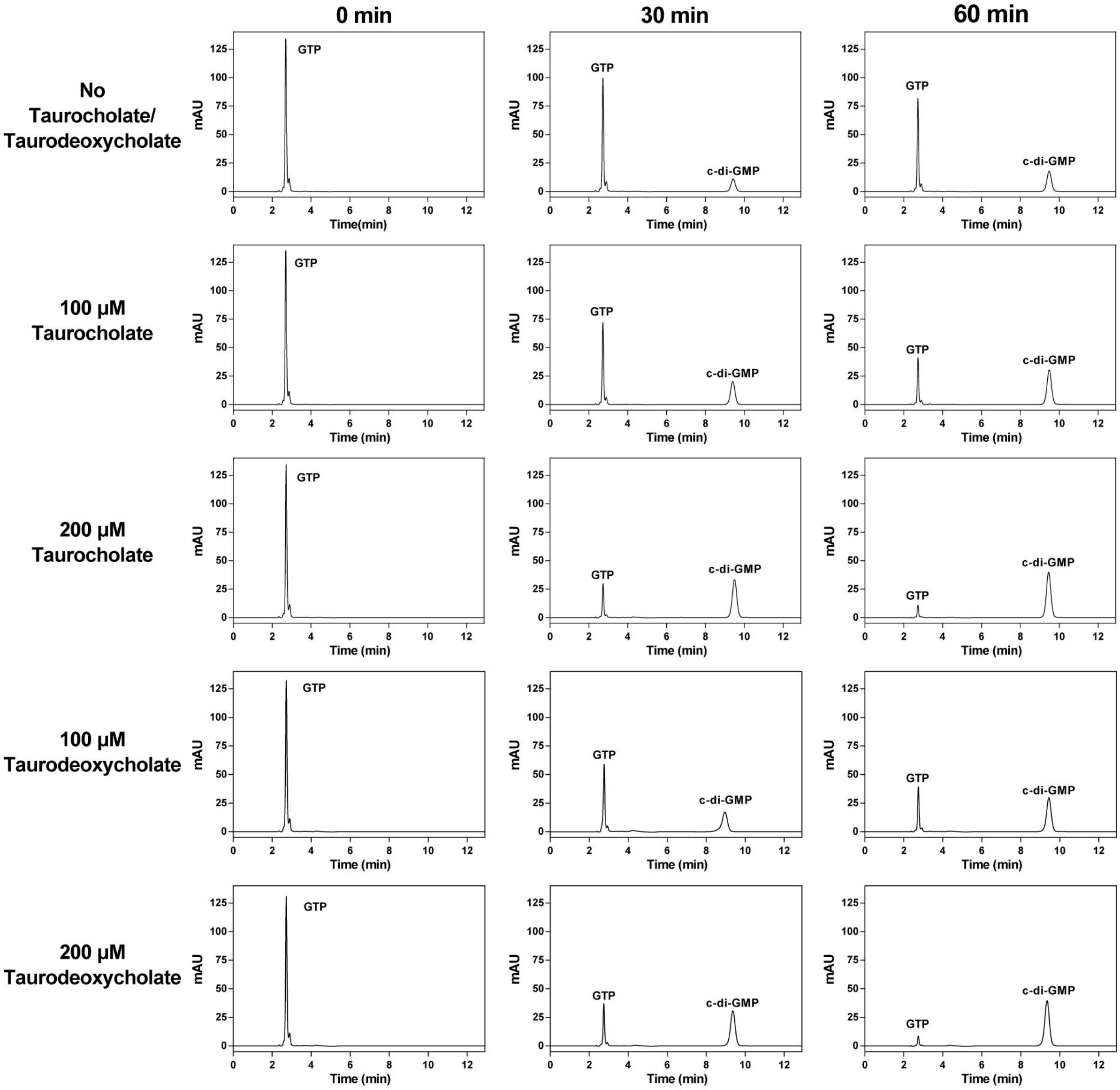

Supplementary Fig. 12 Taurocholate and taurodeoxycholate induce the DGC activity of YedQ in c-di-GMP synthesis

Membrane fractions containing full-length YedQ were incubated with GTP in the presence and absence of the bile components taurocholate or taurodeoxycholate at 30°C for 0, 30 and 60 min and then the reaction products were analyzed by HPLC. The shown spectra are representatives of three independent experiments with similar results.

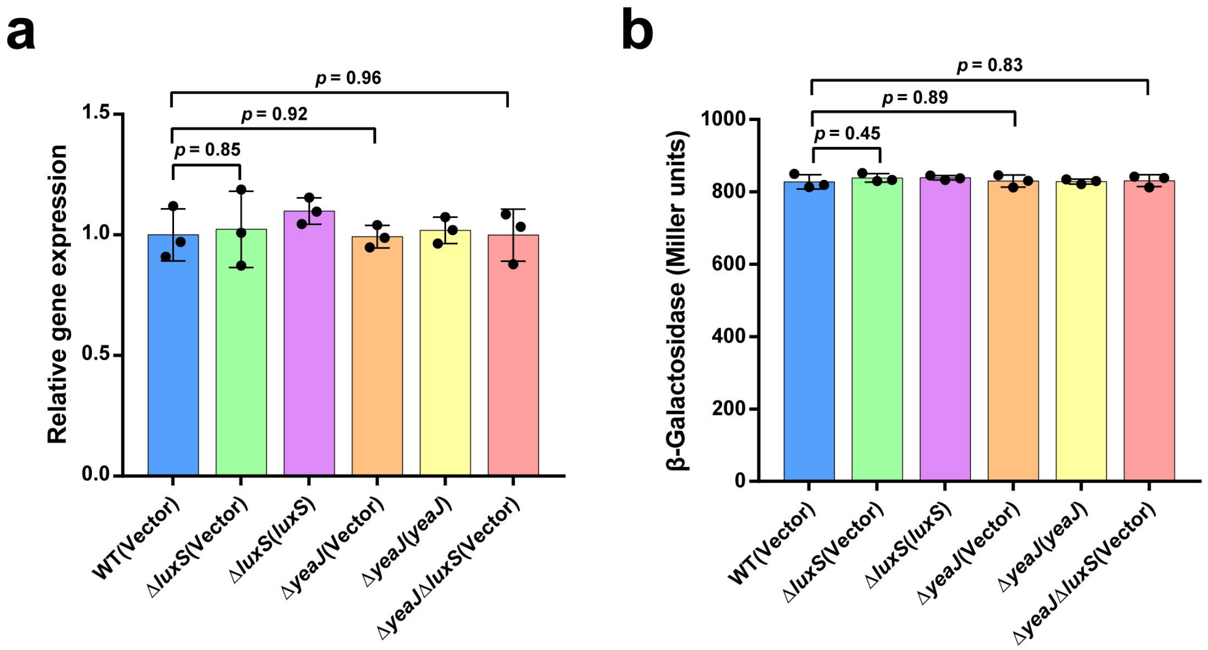

Supplementary Fig. 13 Deletion of *luxS* or *yeaJ* does not affect mRNA levels of *invF* and its promoter activity

Cultures were grown in modified LB medium containing 0.3 M NaCl at 37 °C without agitation to an OD_{600} of 1.1, and then harvested for qRT-PCR analyses (**a**) and promoter activity assays (**b**). The level of *invF* gene expression was normalized to 16S rRNA and reported as fold change relative to that of the wild type. Promoter activity of *invF* was determined by quantifying β-galactosidase activity. Data are mean ± s.e.m. of three independent experiments. Statistical significance was determined by two-tailed Student's *t*-test. $P < 0.05$ indicates that the difference is statistically significant. Source data are provided as a Source Data file.

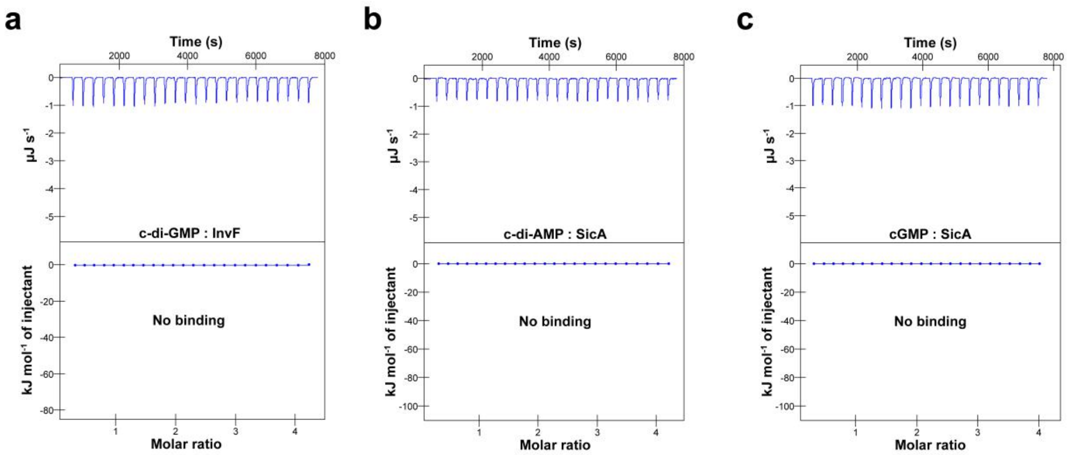

Supplementary Fig. 14 Isotherms representing binding of InvF with c-di-GMP and SicA with c-di-AMP and cGMP, measured by ITC

ITC data and plots of injected heat for injections of 100 μM c-di-GMP (**a**), c-di-AMP (**b**) or cGMP (**c**) into the sample cell containing 10 μM InvF (**a**) or SicA (**b**, **c**) are shown in the upper and lower plots, respectively. The heats of ligand dilution were subtracted from the heats of injection and the corrected data were fit to a one-site binding model. Data shown are one representative of three independent experiments with similar results.

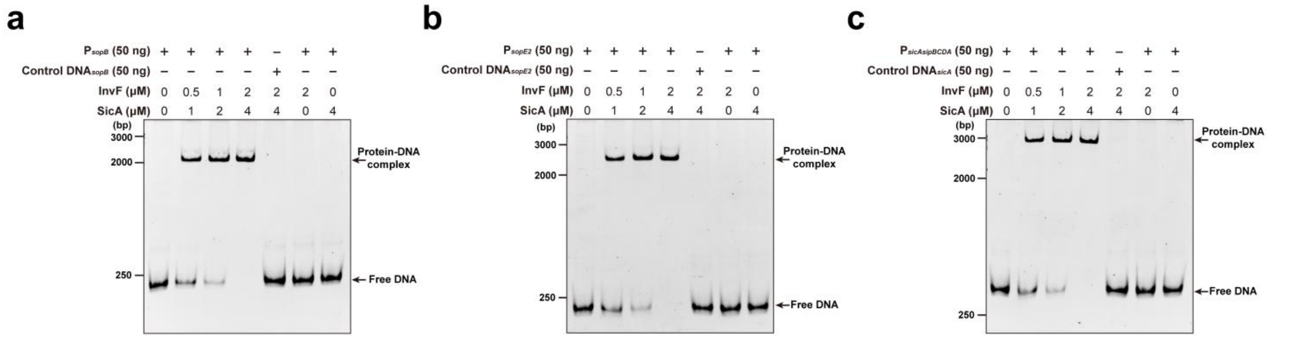

Supplementary Fig. 15 EMSAs demonstrating specificity of InvF/SicA binding to promoters of *sopB*, *sopE2* and the *sicAsipBCDA* operon

50 ng of P_{sopB} (**a**), P_{sopE2} (**b**) or $P_{sicAsipBCDA}$ (**c**) DNA was incubated with InvF and SicA (the concentrations are noted in the panel) in a 20-μl reaction system. DNA fragments amplified from the coding regions of the *sopB* (**a**), *sopE2* (**b**) and *sicA* (**c**) genes were used as controls. Data shown are one representative of three independent experiments with similar results. Source data are provided as a Source Data file.

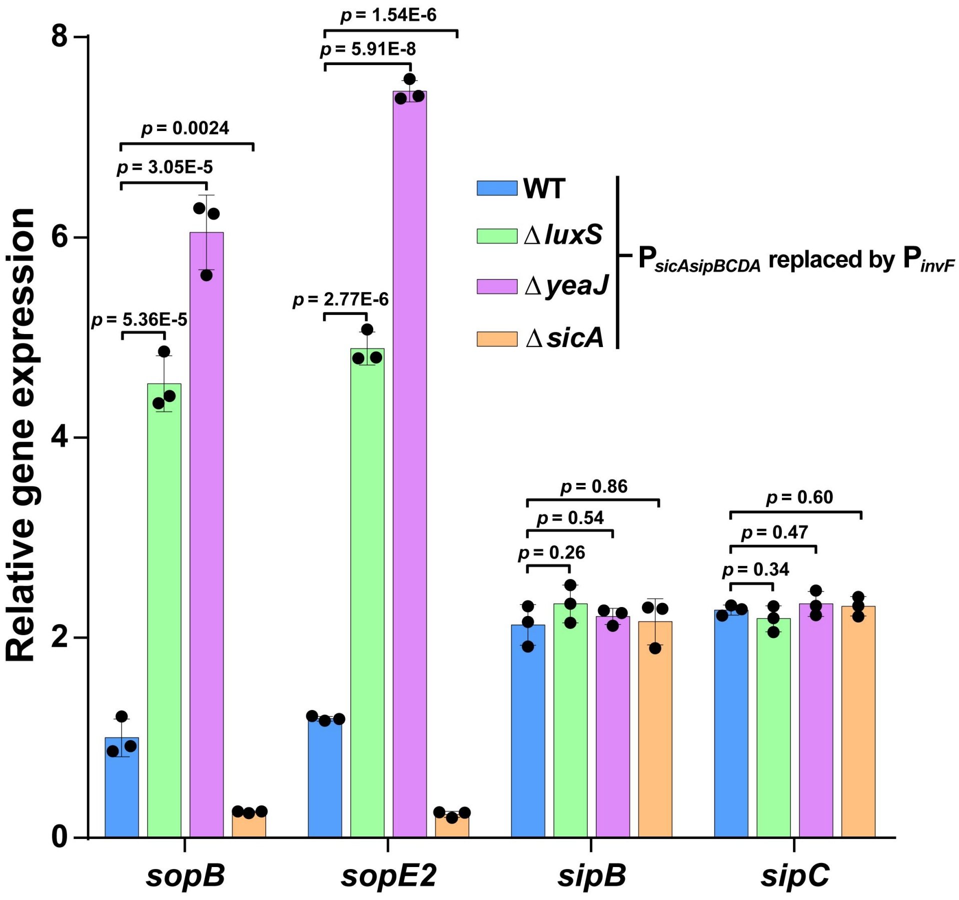

Supplementary Fig. 16 Replacement of the *sicAsipBCDA* promoter with the *invF* promoter in *S.* Typhimurium strains abolishes the response of *sipB* and *sipC* gene expression to the deletion of *luxS*, *yeaJ* or *sicA*

Cultures grown in modified LB medium containing 0.3 M NaCl at 37 °C without agitation to an OD_{600} of 1.1 were harvested for total RNA extraction and qRT-PCR analyses. Gene expression levels were normalized to 16S rRNA and presented as values relative to that of the *sopB* gene of the wild-type (WT) strain with $P_{sicAsipBCDA}$ replaced by P_{invF}. Data are mean ± s.e.m. of three independent experiments. Statistical significance was determined by two-tailed Student's *t*-test. Differences were considered to be statistically significant when *p* values were less than 0.05. Source Data are provided as a Source Data file.

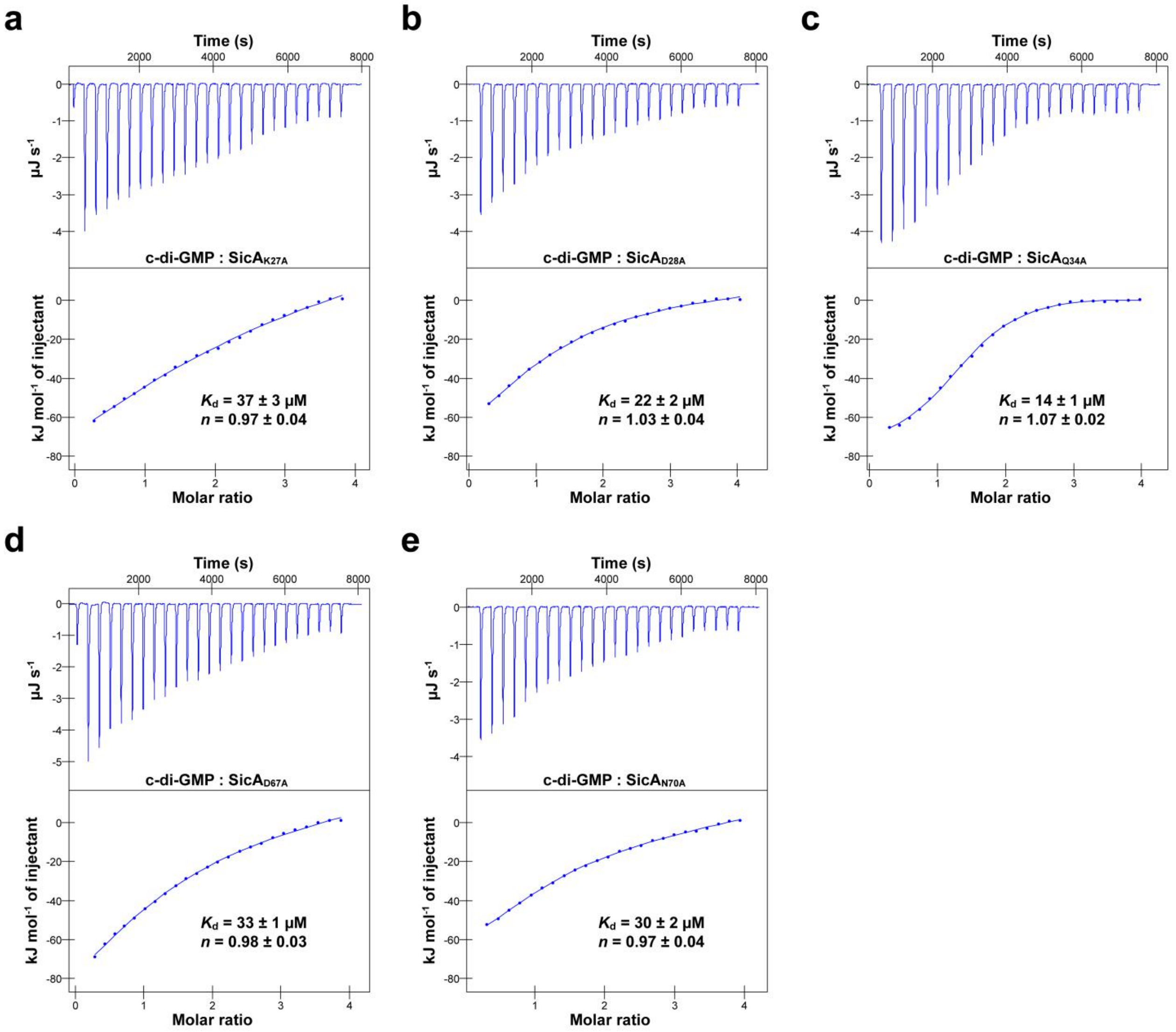

Supplementary Fig. 17 Five mutants of SicA show lower binding affinity to c-di-GMP compared with the wild type

The binding affinity was measured by ITC. ITC data and plots of injected heat for 25 automatic injections of 100 μM c-di-GMP into the sample cell containing 10 μM SicA mutants K27A (**a**), D28A (**b**), Q34A (**c**), D67A (**d**) or N70A (**e**) are shown in the upper and lower plots, respectively. The binding curves were corrected for the dilution effects in the final analysis. Isotherms shown are one representative of three independent experiments and the K_d and binding stoichiometry (n) values were presented as mean ± s.d. of the three independent experiments. Source data are provided as a Source Data file.

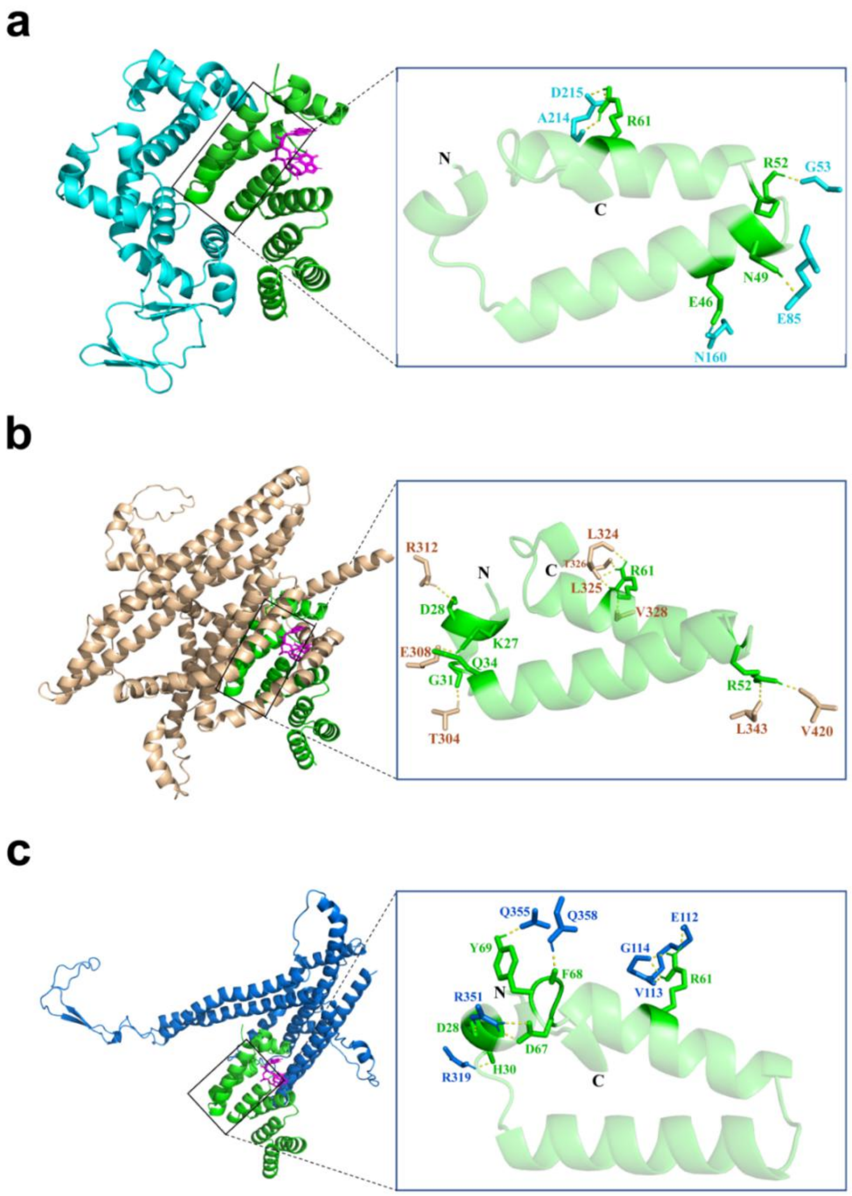

Supplementary Fig. 18 Docking analysis of SicA with InvF, SipB and SipC

A homology model of SicA was constructed via the Phyre2 server based on the crystal structure of IpgC from *S. flexneri* (PDB ID: 3GYZ; https://www.rcsb.org/structure/3GYZ). The 3D structures of InvF, SipB and SipC were predicted by AlphaFold2. Docking simulation of SicA with InvF (**a**), SipB (**b**) and SipC (**c**) was performed using the Cluspro 2.0 web server (https://cluspro.org). In left panels, cartoon representations of the SicA (green) in complex with InvF (cyan), SipB (gold) and SipC (blue) are shown. Right panels depict interactions of SicA (residues 25-72) with InvF, SipB and SipC, and residues important for binding are shown in sticks with hydrogen bonds indicated by yellow dashed lines. C-di-GMP is also shown as pink sticks in the predicted c-di-GMP binding site of SicA as indicated in Fig. 5i.

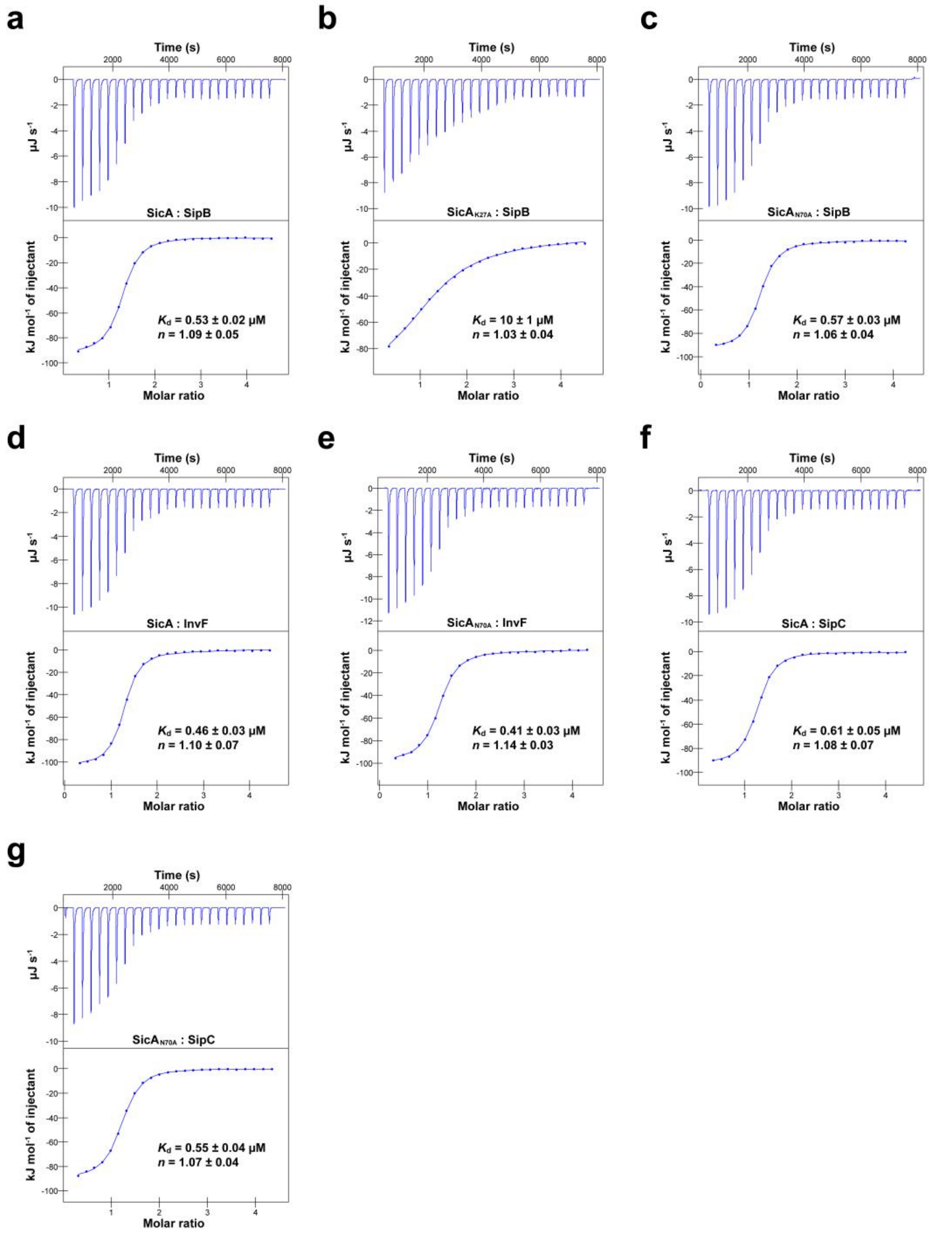

Supplementary Fig. 19 Isotherms representing binding of SicA and its variants with InvF, SipB and SipC as measured by ITC

ITC data and plots of injected heat for 25 automatic injections of 100 μM SicA (**a, d, f**), $\text{SicA}_{\text{K27A}}$ (**b**) or $\text{SicA}_{\text{N70A}}$ (**c, e, g**) into the sample cell containing 10 μM SipB (**a-c**), InvF (**d, e**), or SipC (**f, g**) are shown in the upper and lower plots, respectively. The binding curves were corrected for the dilution effects and fit to a one-site binding model. Isotherms shown are one representative of three independent experiments with similar results. The K_d and binding stoichiometry (n) values are shown as mean ± s.d. of three independent experiments. Source data are provided as a Source Data file.

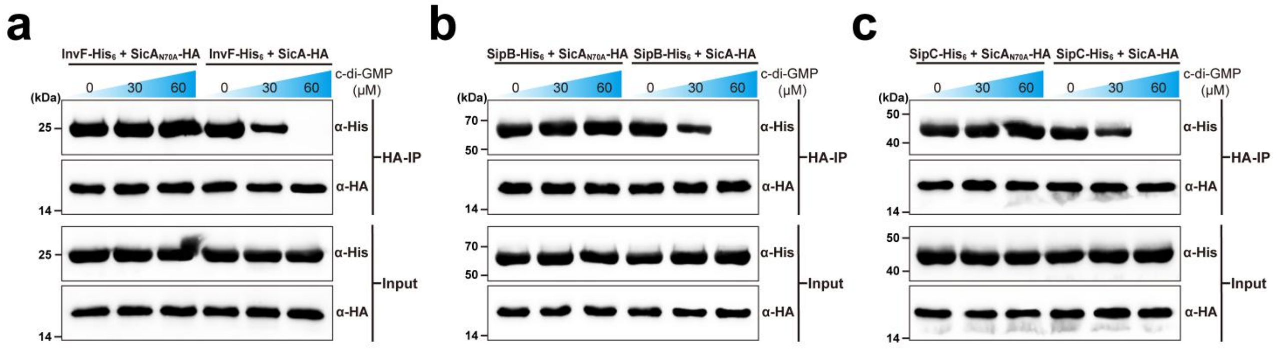

Supplementary Fig. 20 C-di-GMP does not impair co-IP of InvF-His$_6$ (a), SipB-His$_6$ (b) and SipC-His$_6$ (c) with SicA$_{N70A}$-HA

S. Typhimurium strain SL1344 producing both HA-tagged and His$_6$-tagged proteins were grown at 37 °C in LB medium to an OD$_{600}$ of 2.0, and then bacterial cells were collected, lysed and immunoprecipitated using beads coupled to anti-HA antibodies in the presence and absence of c-di-GMP (0, 30 or 60 μM). The immunoprecipitated proteins (HA-IP) and the total cell lysates used for co-IP (Input) were assessed by western blot with anti-HA or anti-His antibodies. Blots shown are one representative of three independent experiments with similar results. Source data are provided as a Source Data file.

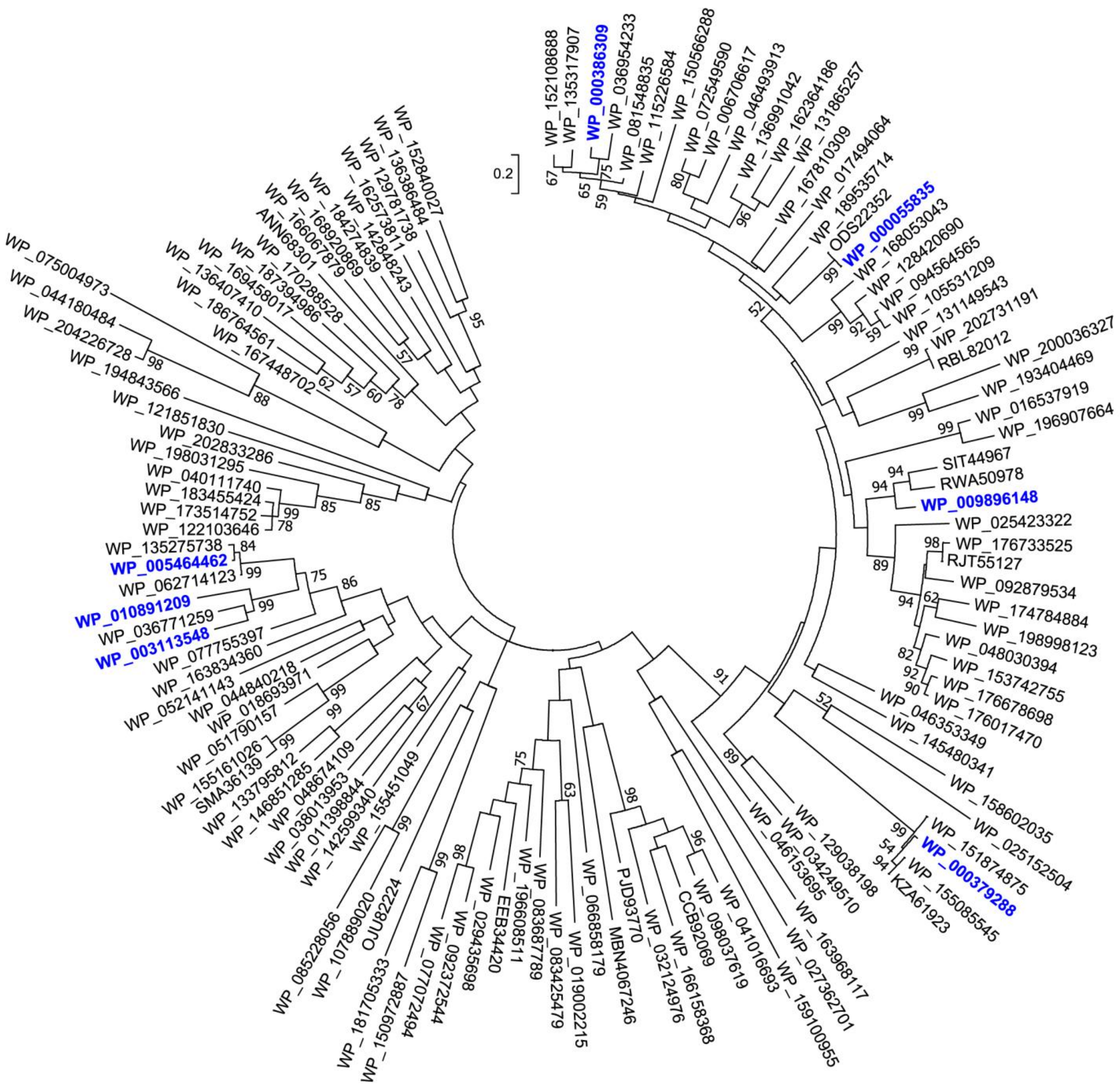

Supplementary Fig. 21 Maximum-likelihood phylogenetic tree showing the relationship of the T3SS-1 chaperone SicA from *S.* Typhimurium and its homologs from other genera

SicA homologs were searched by BLASTP against the NCBI non-redundant protein database with a cutoff E-value of 1E-03 and a coverage threshold of 60% (Supplementary Data 2). One representative sequence for each genus was randomly selected and multiple sequence alignments were performed with clustalX version 1.81. A maximum-likelihood phylogenetic tree was constructed based on the Jones-Taylor-Thornton (JTT) model with bootstrap values determined by 1,000 replicates in the MEGA7 software. The NCBI accession numbers for SicA homologs are shown and their taxonomy affiliations are provided in Supplementary Data 2. SicA (WP_000386309) and its homologs from the phylum *Proteobacteria* that have been found to bind c-di-GMP are indicated in blue. Bootstrap values greater than 50% are shown on the branch. Scale bar indicates evolutionary distance of 0.2 amino acid substitutions per position.

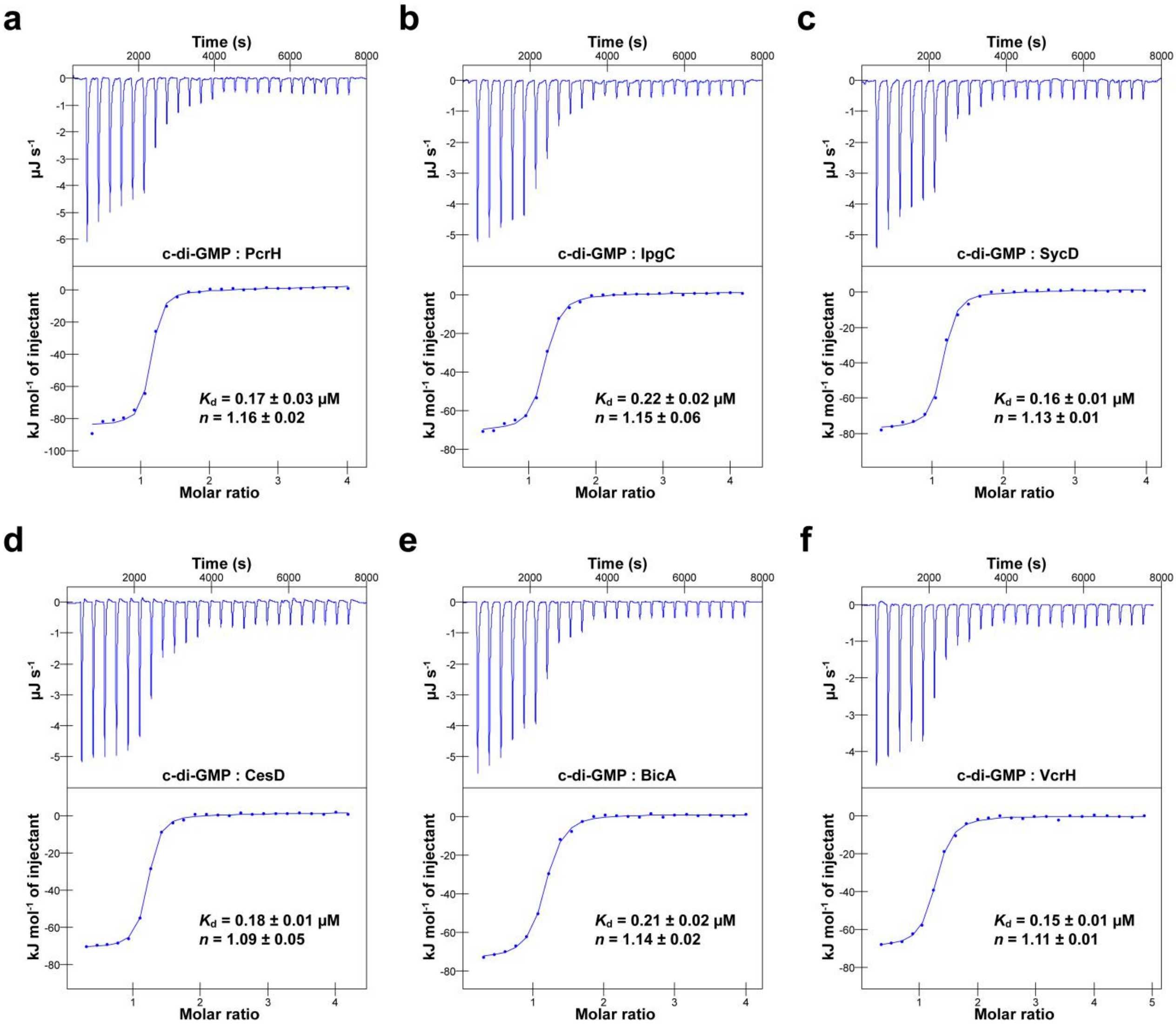

Supplementary Fig. 22 SicA homologs from six pathogenic bacteria belonging to the phylum *Proteobacteria* show high binding affinity to c-di-GMP

The binding affinity was measured by ITC. ITC data and plots of injected heat for 25 automatic injections of 100 μM c-di-GMP into the sample cell containing 10 μM PcrH (NCBI accession number WP_003113548) of *P. aeruginosa* (**a**), IpgC (WP_000055835) of *S. flexneri* serotype 2a (**b**), SycD (WP_010891209) of *Y. enterocolitica* (**c**), CesD (WP_000379288) of EHEC O157:H7 (**d**), BicA (WP_009896148) of *B. thailandensis* (**e**) or VcrH (WP_005464462) of *V. parahaemolyticus* (**f**) are shown in the upper and lower plots, respectively. The binding curves were corrected for the dilution effects in the final analysis. Isotherms shown are one representative of three independent experiments with similar results. K_d and binding stoichiometry (n) shown are mean ± s.d. of three independent experiments. Source data are provided as a Source Data file.

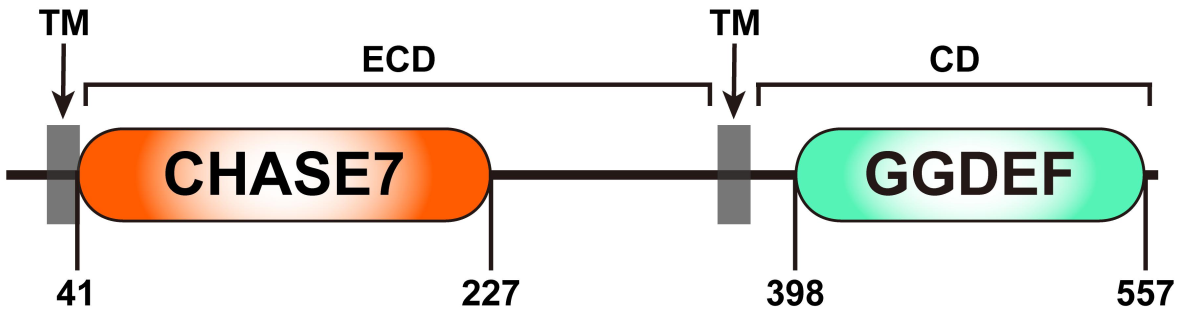

Supplementary Fig. 23 Schematic illustrating the predicted domain organization of YedQ

Domain architecture for YedQ from *S*. Typhimurium strain SL1344 was predicted using hmmscan against the Pfam 34.0 database. The gathering threshold is used to determine hit significance. TM, transmembrane domain; ECD, extracytoplasmic domain; CD, cytoplasmic domain.

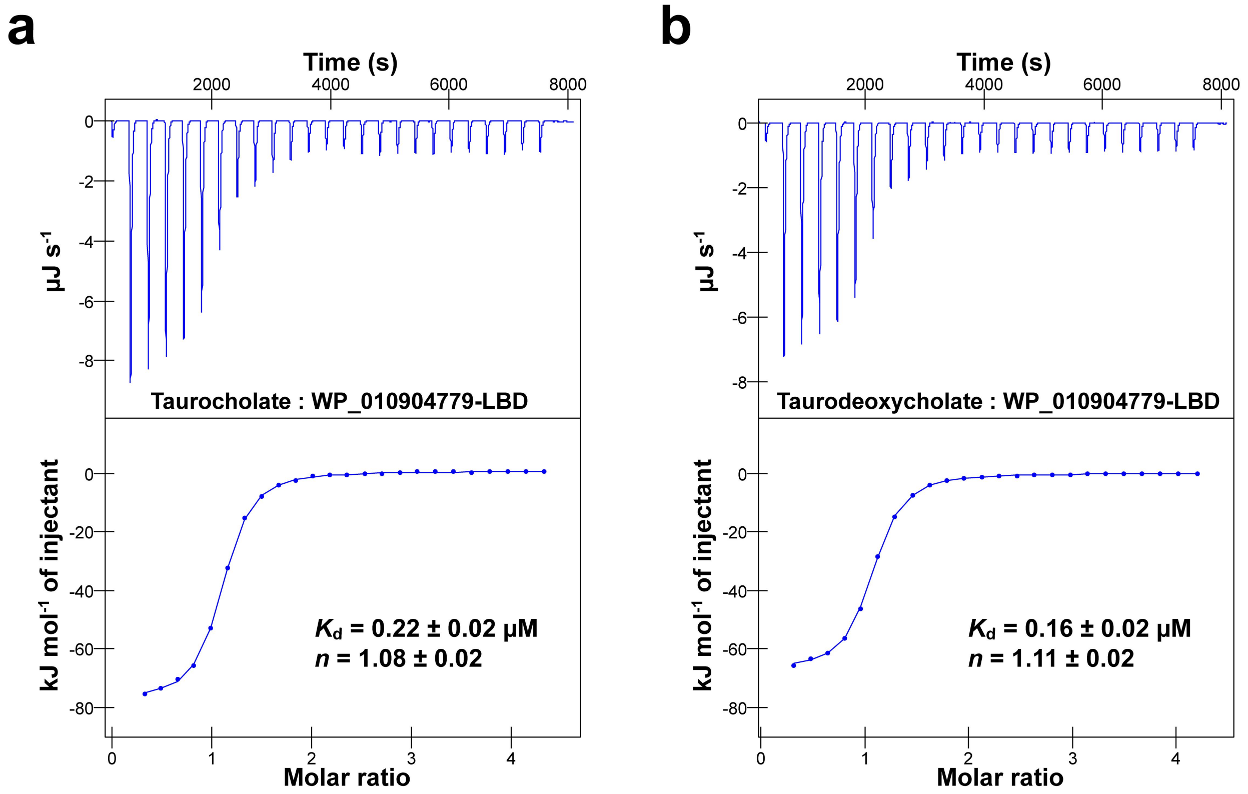

Supplementary Fig. 24 The LBD of the YedQ homolog from EHEC O157:H7 shows high binding affinity to taurocholate and taurodeoxycholate

The binding affinity was evaluated by ITC analysis. ITC data and plots of injected heat for automatic injections of 100 μM taurocholate (**a**) or taurodeoxycholate (**b**) into the sample cell containing 10 μM WP_010904779-LBD are shown in the upper and lower plots, respectively. The binding curves were corrected for the dilution effects in the final analysis. Results shown are one representative of three experiments with similar results. K_d and binding stoichiometry (*n*) are presented as mean ± s.d. of three independent experiments. Source data are provided as a Source Data file.

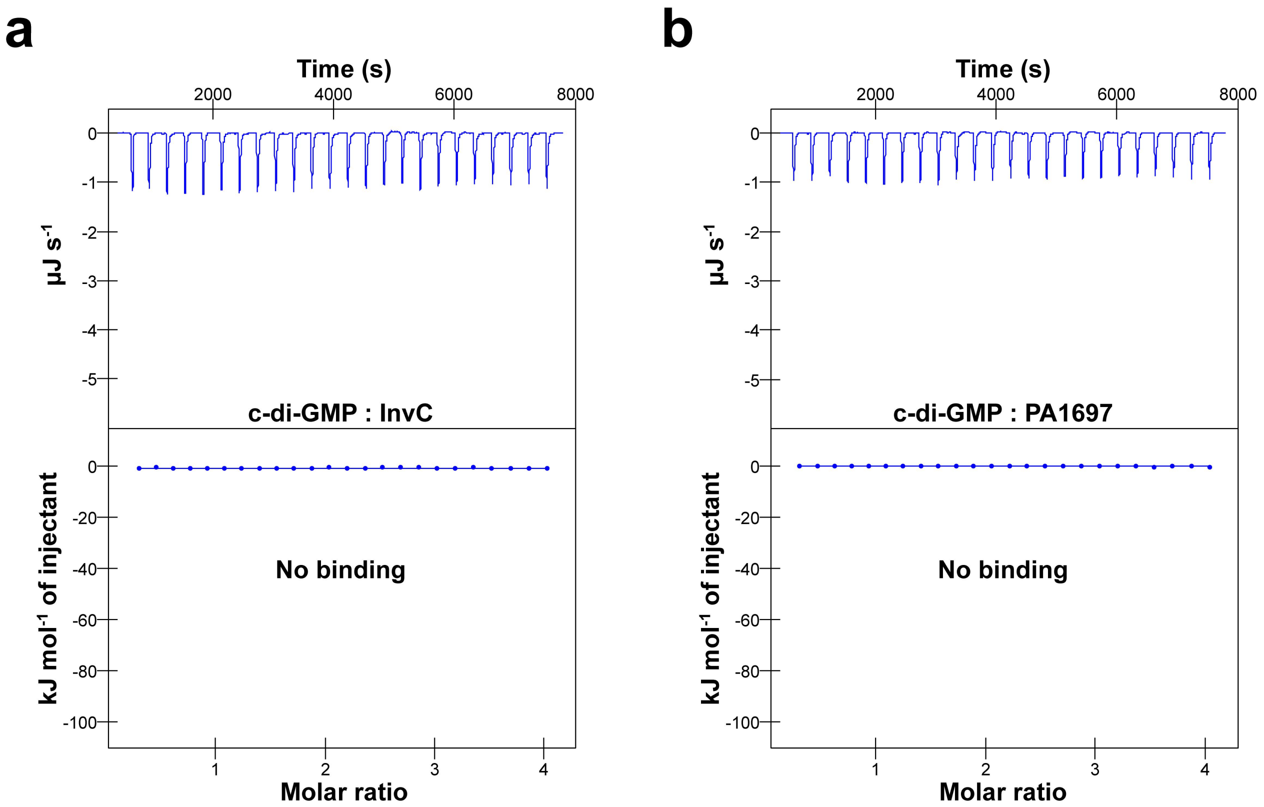

Supplementary Fig. 25 Neither the T3SS ATPase InvC (STM2894) from *S*. Typhimurium nor the T3SS ATPase PA1697 from *P. aeruginosa* interacts with c-di-GMP

The binding affinity was evaluated by ITC analysis. ITC data and plots of injected heat for automatic injections of 100 μM c-di-GMP into the sample cell containing 10 μM InvC (**a**) or PA1697 (**b**) are shown in the upper and lower plots, respectively. The binding curves were corrected for the dilution effects in the final analysis. Results shown are one representative of three experiments with similar results.

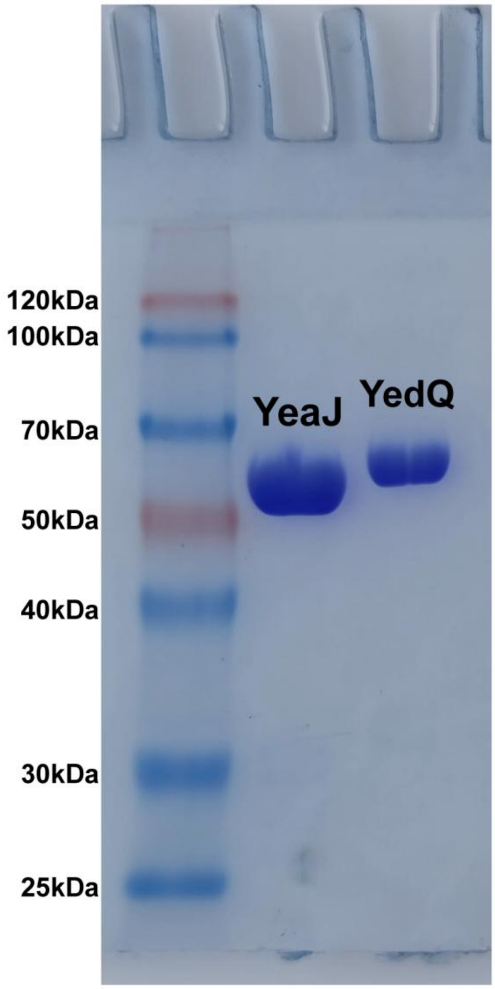

Supplementary Fig. 26 SDS-PAGE gels of purified full-length YeaJ and YedQ from *S.* Typhimurium

After inverted membrane extraction and further purification by Ni^{2+}-NTA affinity chromatography, membrane fractions containing full-length YeaJ and YedQ were subjected to SDS-PAGE analysis to examine the purity. Similar results were obtained in three independent experiments.
