# Supplementary material for: Autoinducer-2 and bile salts induce c-di-GMP synthesis to repress the T3SS via a T3SS chaperone
Source: Nat Commun. 2022 Nov 5;13:6684. doi: 10.1038/s41467-022-34607-9 (PMC9637222; doi:10.1038/s41467-022-34607-9)
Supplement: Supplementary file 3 — Description to Additional Supplementary Information [file 41467_2022_34607_MOESM3_ESM.pdf]

Description of Additional Supplementary Files

Supplementary Data 1 YeaJ homologs that possess a putative N-terminal GAPES1 domain and a putative C-terminal GGDEF domain.

Searches for YeaJ homologs were performed using the BLASTP program against the NCBI non-redundant protein database with a cutoff E-value of 1E-03 and a coverage threshold of 60%. Sequences were further analyzed using InterProScan 5 against the Pfam 34.0 database, and only the sequences that possess a N-terminal GAPES1 domain and a C-terminal GGDEF domain with a cutoff E-value of 1E-10 were remained. NCBI accession numbers, pairwise Identity (%) and query coverage (%) with YeaJ, protein length, Pfam domain architectures, output domains, predicted protein function, the organisms from which the YeaJ homologs were taken and their taxonomy are listed. For each domain, the starting and ending position in protein, domain length and domain E-value are given.

Supplementary Data 2 Putative SicA homologs that share >20% sequence identity with SicA.

Searches for SicA homologs were performed using the BLASTP program against the NCBI non-redundant protein database with a cutoff E-value of 1E-03 and a coverage threshold of 60%, and only putative SicA homologs that share >20% sequence identity with SicA were remained. NCBI accession numbers, pairwise Identity (%) and query coverage (%) with SicA, protein length, predicted protein function, the organisms from which the SicA homologs were taken and their taxonomy are listed.

Supplementary Data 3 Bacterial strains and plasmids used in this study.

Bacterial strains and plasmids used in this study, their relevant characteristics and sources are listed.

Supplementary Data 4 Primers used in this study.

All primers used in this study were designed using Primer premier 5.0 (Premier Biosoft) and synthesized by Tsingke Biological Technology (Xi'an, China). Sequences of the primers are listed, with underlined sites indicating restriction enzyme cutting sites added for cloning.
